# Ongoing Challenges of Laser-Based Powder Bed Fusion Processing of Al Alloys and Potential Solutions from the Literature—A Review

**DOI:** 10.3390/ma16031084

**Published:** 2023-01-26

**Authors:** Alessandra Martucci, Alberta Aversa, Mariangela Lombardi

**Affiliations:** 1 Department of Applied Science and Technology, Politecnico di Torino, Corso Duca degli Abruzzi 24, 10129 Turin, Italy; 2 Consorzio Interuniversitario Nazionale per la Scienza e Tecnologia dei Materiali (INSTM), Via G. Giusti 9, 50121 Florence, Italy

**Keywords:** additive manufacturing, PBF-LB/M, al-based alloys, ongoing challenges, processability

## Abstract

Their high strength-to-weight ratio, good corrosion resistance and excellent thermal and electrical conductivity have exponentially increased the interest in aluminium alloys in the context of laser-based powder bed fusion (PBF-LB/M) production. Although Al-based alloys are the third most investigated category of alloys in the literature and the second most used in industry, their processing by PBF-LB/M is often hampered by their considerable solidification shrinkage, tendency to oxidation, high laser reflectivity and poor powder flowability. For these reasons, high-strength Al-based alloys traditionally processed by conventional procedures have often proved to be unprintable with additive technology, so the design and development of new tailored Al-based alloys for PBF-LB/M production is necessary. The aim of the present work is to explore all the challenges encountered before, during and after the PBF-LB/M processing of Al-based alloys, in order to critically analyse the solutions proposed in the literature and suggest new approaches for addressing unsolved problems. The analysis covers the critical aspects in the literature as well as industrial needs, industrial patents published to date and possible future developments in the additive market.

## 1. Introduction

In recent decades, additive manufacturing (AM) has been widely explored in industry and academia and has consequently become a key driver for the fourth industrial revolution [1]. Among AM techniques, the laser-based power bed fusion (PBF-LB/M) process stands out for producing complex-shaped functional components with good surface quality, excellent dimensional tolerance and unprecedented mechanical properties [2]. During this process, a tightly focused laser beam selectively melts a bed made of fine metal powder layer-by-layer achieving extremely high cooling rates (up to 10^5^ K/s) [3]. The portfolio of metallic materials which can be processed via PBF-LB/M is continuously expanding. Among the alloys currently available, Al-based alloys are gaining increasing interest, especially in the automotive and aerospace sectors, thanks to their excellent strength-to-weight ratio and resistance to cyclic loads [4]. Therefore, the PBF-LB/M processing of commercial Al-based alloys and the development of new Al-based compositions are growing areas and, consequently, present numerous challenges. Considering the significant impact of processing quality on the mechanical and fatigue behaviour of final components [5], intense efforts at the research and industrial level need to be devoted to overcoming these challenges.

**Figure 1:**
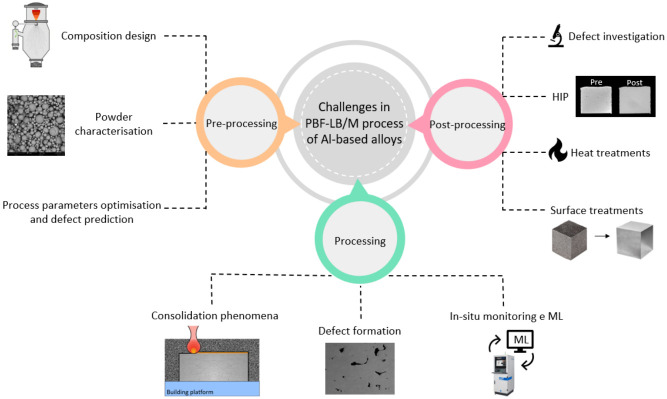
Graphical overview of the review.

The literature reports numerous works describing the major and minor achievements in this area, but often the unsolved or hidden problems of the solutions proposed are not mentioned. In order to fill this gap, the present work has explored and reported the main challenges related to the design and characterisation of Al-based alloys presented in the literature. Since several aspects need to be considered during the development of an alloy, this review paper has divided them into activities occurring pre, during and post-PBF-LB/M production (as summarised in Figure 1).

Concerning the research activities that need to be performed pre-PBF-LB/M processing, the following critical aspects are examined:

- The predictions of solidification behaviour, microstructural features and mechanical performances;
- The procedures concerning powder characterisation and the effects of reuse on powder properties;
- The process parameter optimisation and defect prediction methods.

Looking more closely at the complex phenomena occurring during the PBF-LB/M process, the following critical aspects are described:

- Consolidation phenomena;
- Defect formation mechanisms and defect mitigation approaches;
- Application of machine learning for in-situ process optimisation.

Finally, the following critical aspects post-PBF-LB/M part production are analysed:

- Approaches for defect investigation;
- Post-processing procedure for defect mitigation;
- Heat treatments;
- Surface post-treatments.

A thorough scientific understanding of the challenging aspects of processing Al-based alloys via PBF-LB/M and a critical evaluation of the solutions already identified in the literature are necessary for the development of innovative processing and characterisation approaches. Moreover, a synergetic exploration of the literature gaps and industrial needs may pave the way for extensive industrial use of Al alloys and the PBF-LB/M process. Based on this consideration, the present in-depth review was conducted not only by considering the critical aspects detected in the literature but also by looking at the industrial needs, industrial patents published to date and future developments in the additive market.

## 2. Pre-Processing

### 2.1. Deep Insight into Developing Novel Compositions Tailored for PBF-LB/M

**Figure 2:**
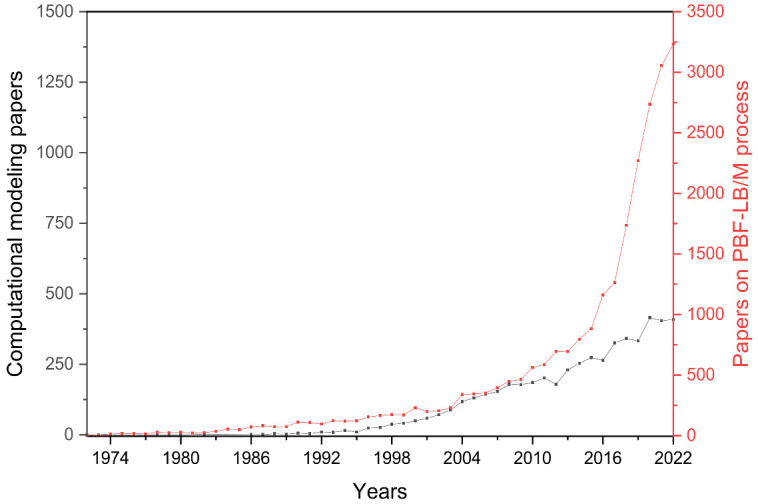
Literature trends on the development of computational models for the novel composition design related to the growth of the PBF-LB/M process, according to the Scopus database.

Although the excellent strength-to-weight ratio of Al-based alloys has attracted broad interest in several industrial fields, their considerable solidification shrinkage, tendency to oxidation, high laser reflectivity and poor powder flowability make these alloys challenging to process by PBF-LB/M [6,7]. To overcome these common drawbacks of Al-based alloy processed through PBF-LB/M, much effort has been dedicated to finding novel tailored Al-based compositions characterised by high heat stability and mechanical performance. However, optimising the composition requires an exhaustive trial and error experimentation that implies significant time and costs. For this reason, developing analytical and computational models for designing and optimising alloy compositions has become one of the most important ongoing challenges in this field. As displayed in Figure 2, according to the Scopus database, interest in developing computational models has moved hand in hand with the growth of the PBF-LB/M process. The graph in Figure 2 emphasises, in fact, that as interest in the PBF-LB/M process grew, the need to develop computational models also grew. This synergy in trends can be explained since the ‘trial and error’ approach appeared unsuitable and extremely cost- and time-consuming for this additive technique, while computational models could represent powerful allies in developing new high-performing alloys for the PBF-LB/M process reducing time- and powder-waste.

Due to the intrinsic difficulty of this task, computer calculations and simulations have been carried out at different length scales, following the integrated computational materials engineering (IMCE) paradigm. Several computer codes are available to describe phase transformations, including melting and solidification, and to compute various properties for different types of materials. Among the more commonly used software, there are commercial ones such as Thermo-Calc [8], JMatPro [9], MatCalc [10], Pandat [11] and open source ones such as OpenCalphad [12] and PyCalphad [13]. These are usually based on the CALculation of PHAse Diagrams (CALPHAD) method, which can describe equilibrium thermodynamics and phase diagrams of complex multicomponent systems. Despite the many developed software and the remarkable progress achieved in the literature on this topic, the problems listed below are still unsolved.

- Thermophysical data availability
- Selection of solidification models suitable for the PBF-LB/M process
- Reliability in predicting alloy processability and mechanical properties

Regarding the thermophysical data availability, although reference data have been evaluated for almost all pure liquid metals [14], for liquid alloys and, in particular, for high melting point alloy compositions, such information is often scarce or unavailable. For example, Mohr et al. [15] derived the thermophysical properties of their alloy using containerless electromagnetic levitation on the International Space Station. Following the CALPHAD method, a multicomponent database is usually built starting from low-order subsystems (binaries, ternaries, etc.). Nevertheless, the properties of each subsystem need to be evaluated or taken from the literature [16]. On the other hand, at the same time, the experimental data remain necessary for the validation of theoretical models [17].

The second main challenge lies in the models used for simulations of solidification mechanisms. Based on thermodynamic databases, in equilibrium conditions, the solidification of an alloy can be simulated by minimising the total Gibbs energy of the system using the Lever-rule model [18]. However, in the PBF-LB/M process, a far-from-equilibrium solidification with the formation of metastable phases is induced due to the rapid cooling rates above 10^5^ K/s [19]. The non-equilibrium solidification simulations are usually carried out using the Scheil–Gulliver model, which assumes infinite diffusion of the elements in the liquid phase, no back diffusion in the solid phase and thermodynamic equilibrium at the interface. Although Bocklund et al. [20] demonstrated that the phases predicted by the Scheil–Gulliver model agree better with the experimental results than the predictions made by assuming equilibrium solidification, the assumptions of this model represent an evident theoretical limit. In particular, less segregation arises during real PBF-LB/M solidification processes compared to the Scheil–Gulliver model because diffusion in both liquid and solid phases contributes to mass redistribution. To overcome this theoretical limit, some back diffusion, coarsening, and undercooling considerations were studied to implement Scheil–Gulliver model corrections and achieve a better correlation with experimental results [21]. A synergic approach was attempted by Keller et al. [22] modelling on three levels: Scheil–Gulliver, DICTRA (a software based on a one-dimensional model for diffusion-controlled phase transformation often used to study the micro-segregation phenomenon), and phase-field (a method used to simulate microstructural features through a scalar value order parameter to indicate the presence or absence of a phase). Despite best efforts, many discrepancies between experiments and simulations remain to be resolved. In particular, while a combination of experiments and simulations can successfully predict the phases formed due to the additive process, their effect on the microstructure can only be assumed and not accurately predicted with models [23].

The last major challenge in the field that the authors would like to report is the low accuracy that CALPHAD methods have in predicting the PBF-LB/M processability and the final mechanical properties of a novel alloy without recurring to expensive and time-consuming experimentations. In recent years, models were not merely used to optimise the starting compositions, but their use has extended also to the prediction of PBF-LB/M processability and mechanical properties. A problem that dangerously afflicts the Al-based alloy processability and needs to be considered during the design of novel compositions is their susceptibility to cracking. Among others, Zhang et al. [24] and Kou et al. [25] used the CALPHAD method to predict susceptibility to cracking of different alloys. However, as demonstrated by Zhang et al. [24], back diffusion in the solid phase can significantly exacerbate the cracking susceptibility. This consideration suggests that the prediction of crack susceptibility via CALPHAD methods is subjected to a high degree of uncertainty based on the previously mentioned lack of a model that accurately describes the back diffusion during a PBF-LB/M process. In addition, CALPHAD methods are also characterised by a low accuracy in predicting mechanical properties. As stated by Thapliyal et al. [23], correctly predicting the reinforcement mechanisms underlying the mechanical properties on a purely simulative basis can be challenging. For example, mechanical properties are highly correlated to grain size, but this value can only be approximately defined based on simulation results and assumptions that consider potential inoculant properties of the formed phases extracted from the literature. Until now, only by adding appropriate considerations of grain size distribution and reinforcement contributions to the simulation results is it possible to obtain predictions of mechanical properties similar (but not equal) to those obtained by experimentation. This, therefore, remains one of the biggest ongoing challenges.

### 2.2. Powder Characterisation in Virgin and Reused Conditions

PBF-LB/M is a powder bed process in which a powder is spread on a substrate until a powder layer with a controlled thickness is created. Then, a focused laser beam selectively melts some specific areas of the powder layer according to the STL data of the component. Then, the molten material solidifies by transferring the generated heat to the surrounding media. When the solidification of the first layer is completed, the layer-by-layer production continues until the component is entirely built. Due to the PBF-LB/M building procedure, the powder quality and the quality of the spread layer play a key role in process performance and end part properties [26]. Although numerous recent investigations demonstrated that morphological and dynamic powder properties influence the quality of the powder layer, the melting particle kinetics, the surface roughness and density of manufactured parts [27,28,29,30,31], nowadays, there is no univocal standard of AM powders characterisations. The scientific community has yet to agree on the most suitable characterisation techniques and the fundamental properties that powders should have in order to be considered suitable for an additive process. In most cases, only some aspects are studied, comparative and not quantitative studies are performed, and many case studies are not comparable due to incorrect or unmentioned sampling procedures [28]. Powder flow properties and packing density are unanimously considered the two crucial aspects to consider for a successful PBF-LB/M process. However, as argued by Tio and Caio, it is essential to clarify the difference between flow properties and flowability [30,32]. If flow properties are only influenced by the properties of the powder and the interaction between the particles, the term ‘flowability’ must always be linked to the equipment and the way it was tested. The most common methods for testing the powder flow properties are different, such as the Hall flowmeter funnel (ASTM B213-20), static angle of repose (ASTM C1444), and rotating drum and shear cell (ASTM D7891-15). During the PBF-LB/M process, however, the powder feedstock is deposited with a spreading blade, subjecting the particles to localised shearing and compression. Finding a method that tests the flowability of powders under loading conditions as similar as possible to those occurring during the additive process represents a great challenge [26].

Flow properties are influenced by several factors such as particle size distribution (PSD), powder morphology and storage conditions. Based on a comprehensive review conducted by Vock et al. [30] and the most recently published papers, some general rules can be defined for the flow properties:

- increase with decreasing width of the PSD [33,34,35,36];
- improve with coarser particles [33,34,36];
- decrease with increasing moisture content until saturation with liquid [33,37,38,39].

In other words, the powder flow properties are strongly linked with the powder size and morphology. The latter is, in turn, dependent on the powder production methods. For this reason, choosing the proper production method and correctly optimising the powder production process in order to obtain the optimal PSD and particles morphology for good flow properties should be considered one of the most critical points. The atomisation processes with gas or plasma represent the principal methods used for PBF-LB/M powder production thanks to the spherical shape with limited surface roughness of the powder that could be obtained. In addition, these powder production methods permit a narrow PSD to be achieved, increasing the flow properties of powders. However, these powerful production processes require a careful parameter optimisation. On this topic, the available literature is scarce, even on gas atomisation, which is the currently most widespread method. This literature gap can be explained by the multitude of patents published in the last decade related to the gas atomisation process (more than 1000 patents under the keyword ‘gas atomisation’ are present on the PatentInspiration database from 2013 to date) and the limited number of research centres currently engaged in optimising parameters for powder production. Nevertheless, considering the increasing number of research centres equipping themselves with lab-scale gas atomisers and the key role of these production processes, it will be necessary for the next few years to perform and share investigations in this context.

**Figure 3:**
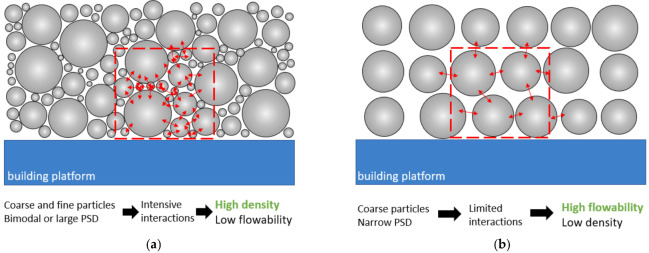
The best scenario for the highest powder packability (**a**) and the best scenario for powder flowability (**b**).

In addition to flow properties, a key role in the PBF-LB/M process is played by the densification and homogeneity of the powder layer. According to a large body of literature, packing density affects a crucial parameter for the PBF-LB/M process, the powder layer height (also known as real layer thickness). The latter is, in fact, defined as the levelling height of the construction platform divided by the percentage of the powder bed effectively occupied by the particles (based on the packing density value). The experimental and simulation studies conducted on the influence of PSD on layer density concluded that a broad PSD or a bimodal PSD with a good fine particle fraction facilitates the obtainment of a dense powder layer. In these cases, the large particles take up the bulk of the space while the smaller particles fill in the gaps left between them [36,40]. However, as illustrated in Figure 3a, this scenario is in contrast to the optimal conditions of flowability, since the fine particles have high surface-to-mass ratio and consequently many van der Waals forces causing the agglomeration and worsening the flowability [40]. On the other hand, when the PSD is narrow and shifted to larger particle sizes, flowability is promoted (Figure 3b). So which PSD represents the right compromise between flowability and packing density? How much fine powder fraction is desirable? Considering the poor flowability of the Al powders due to their low density, these questions need to be addressed.

**Figure 4:**
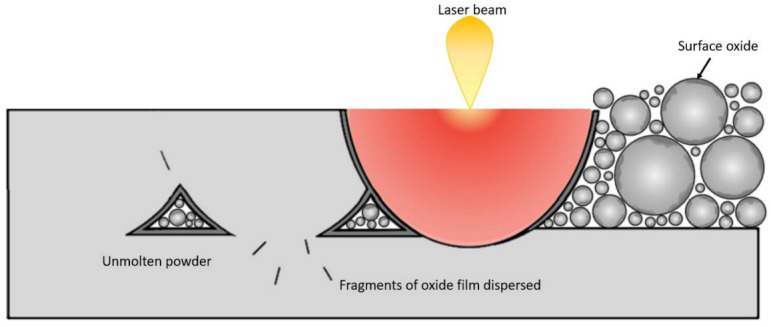
The mechanism of oxide film formation, its disruption and resulting defects.

Defining the basic characteristics needed to assess powder quality and determining quantitative standards is an even greater challenge when considering powder reuse. The powder reuse is a hot topic in sustainability and economic value of additive manufacturing. In fact, the reuse of powders allows remarkable economic and energy savings considering the complicated powder production methods. However, this sustainable practice also has the drawback of increasing the presence of satellites and surface roughness, changing the PSD and forming hydroxides and oxides [41]. The two latter cause powder degradation with a severe impact on the as-built microstructure and mechanical properties of PBF-LB/M produced parts [41,42,43,44]. If the issues concerning the powder morphology and PSD are the same as those related to the characterisation of virgin powders mentioned above, a separate discussion needs to be conducted on the surface chemical changes induced by powder reuse. The tendency of hydroxide and oxide formation is correlated with the high oxygen affinity of the Al matrix and the alloying elements. In fact, Al-based powders tend to oxidise and form Al_2_O_3_ and other oxides depending on the other alloying elements [45,46]. The thermodynamic stability of the oxides and the thermal history of the metal surface determine the characteristics of the surface oxide [47]. In particular, the thickness, structure and composition of the surface oxide are influenced by the temperature reached during the process, the laser exposure time and the level of residual oxygen in the build chamber [48]. During the PBF-LB/M process, the oxide content in the feedstock material results in the formation of an oxide layer on the molten pool that creates a melt pool with a quasi-obtuse contact angle due to the poor wettability resulting in fragments of broken oxide film dispersed in the microstructure (Figure 4) [49]. In addition, the reduced wettability could result in unmelted particles during bulk production, as illustrated in Figure 4 and observed by Read et al. [50] in AlSi10Mg samples. In addition, the inhibition of the local consolidation could lead to the creation of crack formation, drastically reducing the fatigue life of the final component [44].

X-ray photoelectron spectroscopy is considered the most reliable method for the compositional and dimensional evaluation of oxide layers. However, a numerical limit in terms of oxide thickness beyond which the powder cannot be used for the additive process is not yet available in the literature. Instead, several studies report a maximum number of recycling cycles beyond which the powders are too degraded to be used [51]. However, a large part of published papers on this topic cannot be used as a comparison, as the sampling procedure, the building parameters, the size and the geometry of the jobs performed in the recycling cycles are often not reported. Another parameter that influences the powder thermal history but needs to be investigated as it is widely used to reduce residual stresses is the platform heating [52]. Many companies are starting to equip additive machines with heated platforms at increasingly higher temperatures, but their effects on the oxide layer formation and, thus, in the powder degradation after reuse are poorly investigated and deserve further investigation. From the authors’ point of view, a study correlating powder reuse with the bulk-to-powder ratio of the realised jobs should be conducted, as this index inevitably changes the thermal history of the particles.

### 2.3. Process Parameters Optimisation and Defect Prediction

One of the main drawbacks of the PBF-LB/M technique is the incomplete densification that could occur in the final component due to internal defects, such as porosities or cracks [53]. The prominence of internal defects that could cause an early failure of the component is directly linked with the PBF-LB/M parameters [54]. Their optimisation is, therefore, crucial to manufacture dense parts with high mechanical performance. However, Craeghs et al. [55] stated that more than 50 process parameters are involved in the PBF-LB/M process, making the optimisation and monitoring highly challenging. In addition, the process parameters require to be optimised for each powder and PBF-LB/M machine, so it is overly time-consuming.

**Figure 5:**
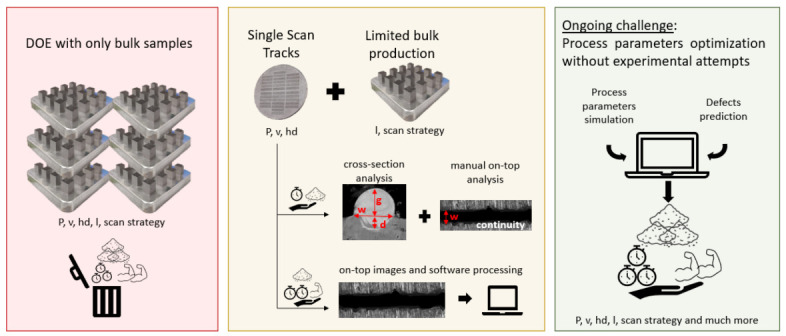
Different process parameter optimisation procedures.

The design of experiments (DOE) approach schematised in the red box in Figure 5, is generally used to systematise the optimisation process. The DOE is a powerful statistical tool that deals with planning, conducting, and analysing controlled tests to investigate the relationship between multiple variables (parameter sets) and key output variables (quality). In PBF-LB/M, the DOE approach usually involves the production of cubic samples with different process parameters and aims to optimise the level of densification. The large number of parameters of the PBF-LB/M process implies that most the work in the literature used the volumetric energy density (VED) for process optimisation. This value embodies the main parameters according to the following equation:

(1) $$VED\;\left(Jmm^{-3}\right)=\frac{P}{v\times hd\times l}$$

where P is the laser power, v is the laser scan speed, hd is the hatch distance and l is the layer thickness.

This approach in which the VED plays a central role is not always the most suitable for the process optimisation. Several factors are involved in the densification phenomena which may be differently influenced by one or more of the parameters embedded in the VED. Among the rare studies providing a comparison between parts produced with constant VED values but under different process conditions, the work of Scipioni et al. [56] stands out. The latter showed the different results that could be obtained by fixing the VED and varying the values of P and v. The relevance of this work lies in having revealed the relevant limitations of using VED as an exclusive design parameter for PBF-LB/M are discussed. The necessity to pay attention to individual PBF-LB/M parameters leads the DOE approach to require a very high number of samples to be produced and analysed, resulting in a time-consuming and powder-wasting procedure.

To streamline the DOE approach, a preliminary process parameter screening was recently introduced, exploiting single scan tracks (SSTs) method (reported in the yellow box in Figure 5) [57]. An SST corresponds to a laser track scanned on a single powder layer previously spread onto a substrate. This approach is based on the idea that PBF-LB/M parts are made of overlapping SSTs, therefore, the properties of the part strongly depend on the geometry of each SST and the interaction among them. To produce SSTs, only P and v can be varied, but a wide range of their combinations can be explored, thanks to rapid production and analysis. Considering the width and the continuity of entire SSTs in an on-top investigation and the cross-section morphologies, a restricted range of P–v combinations suitable for bulk production can be quickly identified [57,58,59]. The SSTs cross-sectional analysis involves a complex and time-consuming sample preparation procedure however the on-top evaluation is strongly affected by the operator error. To numerically evaluate the quality of a SST, an automatic computer-aided method was recently developed by the authors in a previously published work [60]. Although this approach still requires a DOE of bulk samples for full optimisation, a further streamlining of the procedure was enabled by Gheysen et al. [59] and Bosio et al. [61] who developed a method to derive the optimal hd value directly from SSTs. In particular, the optimal hd value can be set by calculating the width of the SSTs and considering an adequate overlapping among them. The latter can be fix at 0% as recommended in Bosio et al. [61] or calculated considering some geometrical on top parameters of the SST using the equation developed by Gheysen et al. [59]. Although the SST approach avoids parameters that would lead to the scan track defects, it cannot fully predict phenomena associated with the layer-by-layer scanning typical of the PBF-LB/M production, as demonstrated by Martucci et al. [62]. This problem is particularly evident with crack-prone Al-based alloys. Therefore, the challenge of optimising the PBF-LB/M process parameters without incurring huge DOEs of bulk samples appears to be not yet completely overcome.

A parameter poorly considered with both optimisation approaches is the layer thickness. In particular, its value is often selected based on the literature of the same alloy family without a proper optimisation procedure. However, some recent studies demonstrated that a correct setting of the layer thickness can allow an impressive increase in the PBF-LB/M productivity by maintaining a good surface finish, an optimal densification and high the mechanical properties of parts [63,64]. This crucial parameter is highly complex to optimise without a large experimental study as it depends on several factors, including the material thermophysical properties, PSD and packing density [65,66]. For this reason, the implementation of an unequivocal rule for calculating the optimum layer thickness considering the materials thermophysical properties, the powders characteristics and the PBF-LB/M machine features remains an ongoing challenge.

Finally, a body of literature has attempted to overcome the challenge to rapidly identify the adequate parameters by combining simulations and experiments to obtain reliable data that can help optimise the production process [67,68,69,70]. Among others, Letenneur et al. [69] evaluated the melt pool dimensions through an analytical model of a thermal field. Then, they experimentally correlated this prediction with the density of the final parts in order to finally link the PBF-LB/M process parameters to densification. Notwithstanding this analytical model provided useful information for the printing of dense parts, it was a simplified model and did not take into account some important information such as the heat exchange and powder spreading conditions [69]. Another recent creditable example is the work done by Maleki et al. [70], who combine deep learning with a stacked autoencoder for prediction and optimization of process parameter using neural networks. However, their approach requires deep knowledge on physical properties of material, which are not easy to find or predict when complex compositions are involved. Therefore, to date, a great deal of work still needs to be carried out on the simulative front to minimise the physical experimentation, as illustrated in the green box in Figure 5.

## 3. During Processing

### 3.1. Consolidation Phenomena

**Figure 6:**
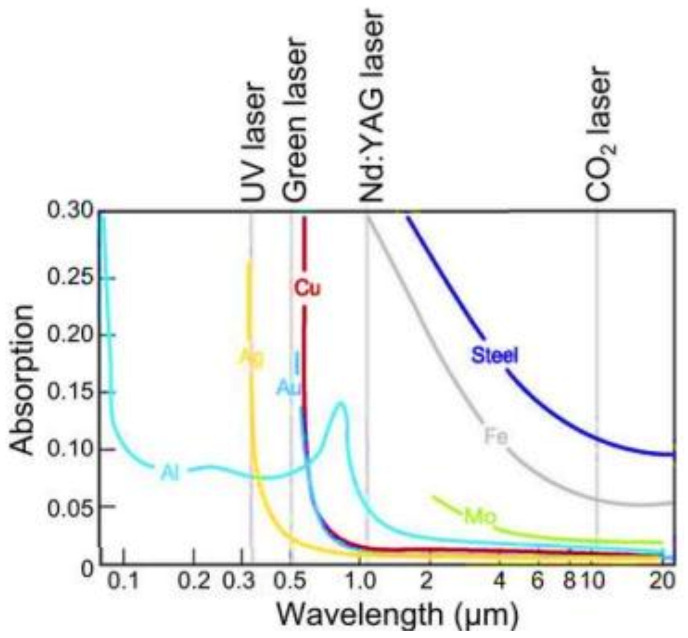
The absorption variation of laser emission at different wavelengths for the main material categories [69].

The absorption rate of a laser beam during a PBF-LB/M process is quite complicated to predict due to the influence of many factors, including the material, the powder surface roughness and the wavelength of the laser. Generally, based on literature results and on the graph reported in Figure 6, the shorter the wavelength, the higher the absorption rate [71].

Looking at the trend of aluminium absorbance as a function of wavelength (in light blue in Figure 6), it is evident that the use of the common YAG laser is a critical aspect in the processing of Al-based alloys by PBF-LB/M due to their low absorption rate and consequent high reflectivity at that wavelength. To mitigate consolidation issues that may occur during YAG laser processing of these alloys resulting in undesired defect formation, very high VED values are usually set. According to the results reported by Prasad et al. [72], a solution could be the use of energy sources at lower wavelengths such as green lasers. In their study, the use of the green laser increased the aluminium absorption more than twofold compared to the use of YAG laser [72]. Despite this evidence, scarce literature can be found on the processing of Al-based alloys with this type of laser. However, the presence over the past five years of several patents on green laser technology (US 8675698B2, US 2022/0314366A1), on converting a laser radiation of longer wavelength to a laser radiation of shorter wavelength (US 9042419B1) and on the use of multi-lasers with different wavelengths (US 2017/0021455A1, US 2015/0246412A1) suggests a clear trend in the PBF-LB/M machine market towards this technology. Prima Industrie S.p.A and Trumpf-Gruppe were pioneers in launching PBF-LB/M machines with green lasers onto the market. The development and commercialisation of these machines will open up new research frontiers and, thus, many new challenges will need to be faced in the coming years.

**Figure 7:**
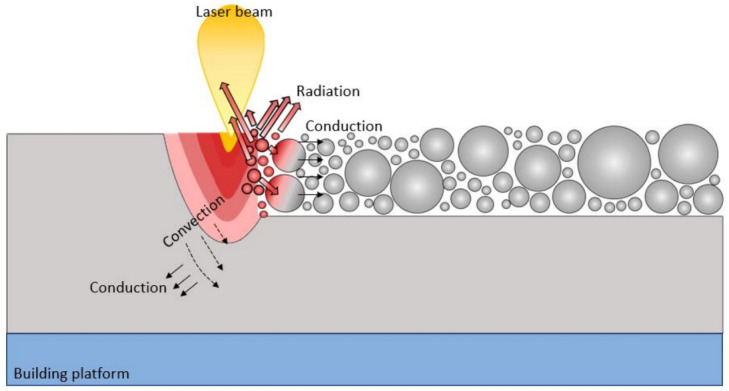
Physical phenomena involved in the PBF-LB/M process.

By modifying the laser absorption conditions, the heat transfer processes of the powder bed are also altered. The heat transfer arises based on three main mechanisms: radiation, the convection with the environment, and conduction within the powder bed and between the powder bed and the substrate, as illustrated in Figure 7.

The PBF-LB/M process is known to have two operating regimes: conduction mode and keyhole mode [73]. Heat conduction is the dominant heat transfer mechanism for fusion in conduction mode, while heat convection is the dominant heat transfer mechanism for fusion in keyhole mode. The condition in which the predominance of conduction or convection depends on the processing conditions is called transition mode. Since these operating regimes are often correlated with the presence of characteristic defects, such as lack of fusion in the conduction mode and keyhole pores in the keyhole mode, their prediction and control are of paramount importance for proper processing by PBF-LB/M. Patel et al. [74], through the careful development of an analytical model in their recent publication, defined that surface vaporisation and a vaporisation depth greater than 0.5 times the beam radius used correspond respectively to the thresholds between conduction and keyhole mode transition for aluminium alloys. However, the robustness of this model on aluminium alloys showed some weaknesses in keyhole mode transition prediction due to the high reflectivity and high thermal conductivity of Al-based alloys. In fact, during the PBF-LB/M processing of Al- based alloys most of the incident beam is reflected, and the melting proceeds more slowly than in low-reflectivity materials (steels or Ti-based alloys) [75]. However, once the front face of the material reaches the melting point and begins to melt, melting proceeds rapidly. Therefore, the transition interval between conduction mode and keyhole mode welding for aluminium is narrow, and the melt pool morphology prediction becomes very sensitive to heat input. Another aspect that makes the applicability of this model challenging is the frequent presence of volatile alloying elements such as magnesium in the Al alloys. Some of the discrepancies in melting thresholds observed in the work of Patel et al. [74] were explained by the presence of such volatile elements which, due to their high vapour pressures, contribute to the development of a keyhole by reducing the heat input required to achieve the keyhole mode. The reflectivity and conductivity characteristics of aluminium powders make the development of increasingly accurate analytical approaches a challenging task to be solved.

Predicting and controlling heat transfer during the PBF-LB\M process is also essential for its influence on the thermally induced residual stresses of the built components. The heat transfer occurring during the PBF-LB\M process is affected by different factors, including the cooling rate, the presence of heated platform and the use of support structures. This influence is often exploited in the literature to reduce residual stresses in PBF-LB\Med components. In particular, platform heating and the use of support structures are usually used to slow the heat flow between the melt layers and the platform, to decrease the thermal gradient and, thus, to reduce thermally induced stresses in the material [76,77]. However, platform heating is not always advisable for low-melting materials such as aluminium alloys, as the high temperatures could result in a heat treatment that potentially change the microstructure. In contrast, the use of support structures in aluminium components are broadly advisable particularly with complex shaped parts. Nevertheless, the software currently available on the market do not provide an accurate estimation of the required support structures as they are affected by the modelling problems mentioned in Section 2.1 and tend to focus more on properly anchoring the part to the platform rather than ensuring proper heat flow. A systematic approach that minimises trial-and-error experiments and ensures a reasonable trade-off between part adhesion and heat flow control still needs to be sought.

The heat transfer phenomena involved in the PBF-LB/M process influence not only the thermally-induced residual stresses but also the final microstructure and, thus, the mechanical properties of the built components. In particular, the direction of the heat flow is responsible for the peculiar microstructure made of elongated grains oriented along the growth direction and thus parallel to the heat flow detected in most of the PBF-LB/Med Al-based alloys [52]. In fact, the directional heat flow typically drives the formation of columnar grain structures along the building direction inside the melt pools [78]. Instead, equiaxial grains with random orientations in line with their growth kinetics are often observed at the melt pool boundary [79]. The directional thermal history and rapid cooling rates result in an unusual heterogeneous and anisotropic microstructure. The microstructure anisotropy negatively affects the mechanical behaviour of the part, especially if multi-axial loads are applied [80]. To identify microstructures able to minimise the anisotropic behaviour, a predictive model of mechanical properties could be useful. Adapting microstructures to meet application-specific goals is one of the major tasks of research in recent years. However, a model for this purpose does not exist, partly due to the challenges associated with simulating the solidification of the complex microstructures of Al-based alloys.

### 3.2. Defects: Solutions to Their Mitigation and Unsolved Problems

**Figure 8:**
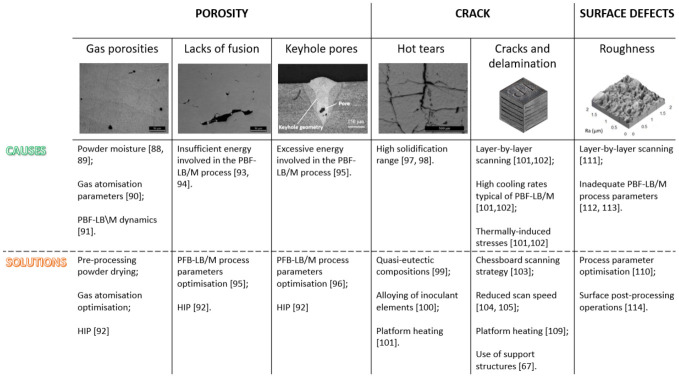
Causes and solutions in the formation of the main defects in the PBF-LB/M process according to the literature.

The high reflectivity and thermal conductivity of Al powders are extremely critical factors, but they are not the only causes of the formation of detrimental defects during the PBF-LB\M process [81]. Galy et al. [82] distinguished four main types of defects observed in the PBF-LB/M processed parts of aluminium alloys: porosity, cracking, low surface quality and anisotropy. The latter was discussed in the Section 3.1. of this paper and will therefore be dropped here. Figure 8 displays the main categories of PBF-LB/M defects, highlighting their causes and approaches to mitigate them.

The ‘porosity’ category includes different kinds of pores including gas porosity, lack of fusion and keyhole porosity. Gas porosities are small spherical porosities predominantly caused by the moisture in the powders, the gas trapped in the particles after gas atomisation and the dynamics of the PBF-LB\M process. Drying the powder immediately before the additive process proved to significantly reduce moisture from the particle surfaces [83,84]. The amount of gas-trapped may instead be minimised by accurately optimising the gas atomisation process [85]. Notwithstanding these mitigation methods, the dynamics of the PBF-LB/M process make gas porosity unavoidable [86]. The protective gas (argon or nitrogen) present in the PBF-LB\M chamber becomes inevitably trapped by the powder flow, enters the melt pool through the Marangoni flow and results in trapped gas bubbles. Although this kind of porosity is commonly not considered detrimental to mechanical performance thanks to their small size and spherical shape, extreme applications, such as in the aerospace field, need to achieve a perfect densification of the component. To achieve this goal, tailored post-processing operations, such as hot isostatic pressing (HIP), need to be defined [87].

In contrast to gas porosities, lack-of-fusion porosities are rather big in size, typically characterised by irregularly shapes and usually located at the melt pool boundary. Lacks-of-fusion are considered detrimental to the fatigue life and mechanical properties of the component due to their morphology with sharp edges that can act as crack initiation points [88]. These defects are mainly caused by insufficient energy involved during the PBF-LB/M process, which results in an incomplete adhesion of the ongoing melt to the surrounding part. As a result, some lack-of-fusion may contain several unmelted particles, further worsening the component performance [89]. However, as demonstrated by Tan et al. [90], lack of fusion defects could be completely avoided by correctly optimising the building parameters.

Last, but not least, a dangerous defect category is that of keyhole pores. If an excessive laser power is involved in the PBF-LB/M process, the temperature of the melt pool might exceed the material boiling temperature, resulting in the material vaporisation. The recoil pressure caused by vaporisation pushes the surface of the liquid downwards, forming a vacuum zone also known as a keyhole. The temperature of the liquid surface around the keyhole fluctuates markedly, generating recoil pressures and forces oriented in different directions and leading to the collapse of the keyhole. Its entrapment in the solidification front results in the formation of undesirable pores in the final component [91]. Although some keyhole porosity formation mechanisms, including keyhole fluctuation, collapse and bubble growth and shrinkage, remain unclear, significant efforts have been made to investigate how mitigate these defects [92].

Looking closer, the second category of defects established by Galy et al. [82], a further distinction needs to be drawn between hot tearing and cracking. The susceptibility to hot tearing is mainly determined by the large solidification range of the alloy [93]. A high difference between solidus and liquidus temperature leads to the formation of long grains during solidification. When the temperature decreases and the solid phase content increases, the remaining molten liquid is trapped in the intercolumnar regions. Upon solidification, a volume contraction occurs due to the solid–liquid phase transition. The induced stresses cause the liquid phase to separate from the solid phase, resulting in hot tears in the intercolumnar regions that could extend through multiple build layers [94]. The mechanisms that could be exploited to suppress the formation of hot tears are different. Firstly, perform composition changes by switching to quasi-eutectic compositions in order to minimise the solidification range [25]. Secondly, add inoculants to the composition in order to obtain a finer microstructure. In fact, grain refinement prevents the formation of long liquid channels along the large columnar grain boundaries during solidification, thus reducing hot tears [95]. Lastly, increase the volumetric energy density or increase the heating temperature of the platform in order to reduce the speed of solidification and to promote the solidification of equiaxed grains. A better filling of the intercolumnar gap with the residual liquid metal can be achieved by forming a hotter and less viscous melt pool and thus reducing the solidification rate [96]. However, Riener et al. [96] demonstrated that the volumetric energy density optimisation can only reduce the hot tearing formation in a high-strength aluminum 6182 alloy and suggested that a stronger effect could be obtained with a platform heating temperature over 500 °C. However, the latter is an extreme temperature condition for low melting Al alloys and could result in undesirable microstructural changes. Therefore, for some compositions, optimising process conditions is not enough to avoid the phenomenon of hot tearing. Based on this consideration, the main ongoing challenge in this defect category is to find effective solutions that do not involve drastic changes in composition.

In the second category of defects, along with hot tearing, it is possible to find cracks and solidification delaminations. The layer-by-layer scanning and high cooling rates typical of PBF-LB/M production result in a high thermal gradient and, thus, in thermally-induced stresses. The stresses developed during the additive process could lead to undesirable cracking and delamination phenomena, seriously affecting the mechanical performance of the final component. The extremely high stresses induced during the additive process on the built component are mainly caused by the large thermal gradients [97]. The two mechanisms of residual stress formation occurring throughout the PBF-LB/M process were extensively discussed in Li et al.’s work [98]. Among the most widely used methods to reduce residual stresses and avoid cracking or delamination, the optimisation of process parameters stands out. According to the Kruth et al.’s simulations [99], the chessboard scanning strategy with a 90° rotation could be an optimal solution to induce an isotropic stress tensor advantageous for residual stresses in the component, avoiding long scan vector lengths and uniformly oriented scan vectors. An important role in reducing residual stress and avoiding delamination formation is played by the VED [100]. For example, reducing scan speed values, Levkulich et al. [101] achieved an enhanced densification behaviour during the PBF-LB\M process and a reduced level of residual stresses. Another approach broadly used is represented by the platform preheating. Buchbinder et al. [102], among others, observed that this procedure can be beneficial in slowing down heat flow and cooling rates reducing the development of residual stresses and, thus, the delamination and crack formation. To achieve similar effects in terms of reducing the thermal gradient, another method involves the use of support structures. Although the residual stresses can be reduced with different strategies, solving the problem of delaminations is complex and not always possible. For this reason, the most successful strategy often involves the synergetic use of all the approaches described above [62].

The third type of defects pointed out by Galy et al. [82] concerns the surface finishing of PBF-LB/M specimens. Li et al. [103] demonstrated that there is a correlation between surface roughness and processing parameters. In particular, they showed that the higher the laser power, the lower the surface roughness, when the scanning speed value is fixed. Despite the many methods attempted in the process parameter optimisation to achieve lower surface roughness, the surface of as-built PBF-LB/M samples are still unsuitable for different industrial applications and post-processing operations are often required in some parts of the components [104,105,106,107]. The topic “surface post-processing operations” will be extensively discussed in Section 4.4.

### 3.3. In-Situ Prediction of Defect Formation and Forward-Looking Machine Learning

The pores formed during the additive process impair the mechanical performance of final components, severely hampering their widespread application. In-situ monitoring allows the formation of defects to be detected in real time, enabling the operator to stop the job, modify some process parameters, and resume component production. Among the in-situ monitoring tools, X-ray technologies have recently gained increasing attention in this field. Exploiting X-ray technologies, the dynamics of spatter [108], pore formation [109], transfer of gas trapped in the powder into the bulk component, keyhole dynamics and keyhole pore formation [110], melt flow [111], solidification and phase transformation [112] may be investigated in real time. In-situ monitoring can also be used to improve the process repeatability by considering any changes in the powder bed [113]. Although the research world is suddenly evolving towards in-situ monitoring, cloud and edge computing still pose significant challenges to the processing and transmission of real-time sensing data due to the high sampling rate and the heaviness of the data. Furthermore, the real research frontier would be to use data from in-situ monitoring as input for machine learning (ML) techniques. The latter is essential to minimise trial-and-error experimentation, overcome the limitations of simulation systems and increase the repeatability of PBF-LB/M processes. Moreover, current machine learning techniques are merely used in a black-box modelling framework, and the results are not explainable and cannot directly compensate for the understanding of process dynamics [114]. Based on these considerations, much effort is required to investigate this field over the next few years. Creating a loop of in-situ monitoring, ML and online parameters readjustment is important not only for research purposes but also for industries with the aim of enabling additive processes for reliable mass production. This industrial interest is also evident from the numerous recent patents granted by the major additive companies. For example, General Electric holds a patent published in 2021 (US2021146480A1) on a diagnostic system of an additive manufacturing machine. This device allows the determination of parameters from in-situ sensors, the continuous comparison of these parameters with threshold values and, based on this comparation, the determination of a failure mode among a plurality of possible failure modes. General Electric also holds a further recently published patent (US2022035358A1) on a diagnostic system with multiple modules. Looking at the patents published by leading additive companies provides a concrete idea of what is to come on the market and thus of new frontiers for research.

## 4. Post-Processing

### 4.1. Defect Investigation in Bulk Samples

**Figure 9:**
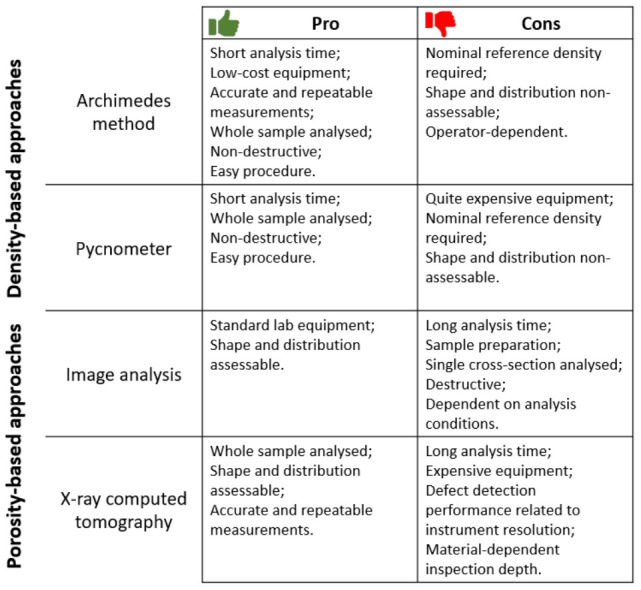
Strengths and weaknesses of the main methodologies for evaluating the level of densification achieved in PBF-LB/M production.

Although the PBF-LB/M process allows parts with a high level of densification to be easily produced, defects cannot be completely avoided. Since defects could seriously compromise mechanical properties acting as crack nucleation sites, the determination of the specimen densification level appears essential. Several methodologies are available to assess the density of a sample, some density-based such as Archimedes method and pycnometer and others defect-based such as image analysis of a cross-section of the sample and X-ray computed tomography (CT). As all these methods have strengths and weaknesses, as described in Figure 9, they are often used synergistically.

Looking closer at the density-based analyses, the Archimedes method stands out for not requiring a long analysis time and any specific equipment. Using the Archimedes method, the sample density can be evaluated from the difference in the buoyancy of an object weighed in air and immersed in a fluid. As demonstrated by Spierings et al. [115], the Archimedes method proved to be accurate and repeatable in absolute density measurements, showing an overall standard deviation for 120 measurements of less than 0.1 %. However, to deduce the part porosity, the evaluated density should be compared to the material nominal reference density. Unfortunately, this latter operation introduces calculation errors, as the material nominal reference density cannot be considered reliable for non-homogeneous parts. Furthermore, as a density-based methodology, it does not allow the shape and distribution of defects to be determined. The other density-based approach to consider is pycnometry, a methodology similar to the Archimedes method but with slightly more expensive equipment. The pycnometer evaluates part density by measuring part volume (determined by gas displacement) and considering the part mass which is evaluated separately. In line with Archimedes, part porosity is derived from the correlation between the evaluated part density and the nominal material density, introducing the same inaccuracy above mentioned. In the pycnometer method, such as in the Archimedes approach, an insufficient accuracy for bulk samples with a high level of densification could be obtained [116]. Moreover, even with this method it is not possible to localise defects or assess their morphology. To achieve this goal, it is necessary to use defect-based methodologies.

As defect-based methodologies, image analysis and CT investigation can be mentioned. The first one could be considered as the most widely employed approach. The image analysis is a destructive method in which samples need to be cut and polished using the standard metallographic procedure before to take several micrographs with an optical microscope. Subsequently, the micrographs require processing using dedicated software which is able to distinguish the pores after thresholding and thus calculate the mean porosity level, the defects distribution and the pores morphology. This methodology is considered accessible, as it merely requires widespread and inexpensive equipment, but the sample preparation and analysis procedure hide several pitfalls. For example, the magnification chosen for the micrographs and the cut plane (parallel or perpendicular to the growth direction) may significantly influence the density measurements. As demonstrated by Spierings et al. [115], the impact of the magnification value and of the analysis plane (horizontal or vertical) on the density measurement is significant for high porosity levels and becomes less remarkable when porosity levels are minimal. In their work, it was proved that the magnification value must be set appropriately to achieve a good compromise between image resolution and the number of images taken, ensuring the identification of even the smallest porosities while having good statistical results. Since samples produced via PBF-LB/M might be characterised by inhomogeneities in the porosity distribution along the growth direction, choosing a plane parallel to the growth direction as the analysis plane is recommended to obtain more representative data [115]. However, inhomogeneities have also been detected in the plane perpendicular to the growth direction, but the literature is not in agreement on this point. For example, Sanaei et al. [117] reported that the highest defect volume fraction and the highest defect concentration is placed near the outer surfaces of specimens. In contrast to this theory there is the research of Spierings et al. [115], where bigger more numerous pores are detected towards the centre of the part. The prediction of defect distribution is highly complex and depends on several factors such as the part orientation, scanning strategy, contour parameters, etc. Since the inhomogeneity of defect distribution is so challenging to predict, examining different sections of the samples is recommended. However, this procedure is more laborious and time-consuming. Another defect-based methodology often described as the best performing is the CT. In line with image analysis, the CT technique permits the investigation of the defect shape, size and distribution. In contrast to image analysis, however, this is a non-destructive technique that allows the entire volume of the sample to be scanned and not merely a section. During a CT scan, the specimen is placed on a rotating stage and irradiated with an X-ray beam. The result is a series of 2D X-ray projections at various angles that are then used to reconstruct a 3D model of the sample with a dedicated software. The reconstruction is in greyscale and a thresholding operation is required to distinguish air from bulk material to transform the 3D reconstruction into binary. From the binary 3D reconstruction, it is possible to extract all morphological and distributional information of the defects present. However, the high cost of equipment and the high operating time remain weaknesses of the CT method. In fact, the minimum size of detectable defects is closely related to the resolution, and high resolutions require long analysis times. Another drawback to point out is the maximum material thickness that can be penetrated by X-rays (Table 1). The latter depends on the material attenuation coefficient and the energy of the X-ray photon. For this reason, although aluminium allows greater thicknesses to be scanned than steel samples, the sample size is still limited.

CT is currently the method that offers the most comprehensive and accurate evaluation of defects; however, many challenges still need to be resolved. In particular, to take full advantage of this powerful technique, increasing the resolution without excessively increasing the analysis time, reducing the heaviness of the data and improving the scanning of multi-material objects could be some of the aspects to be improved.

### 4.2. A Promising Post-Processing Procedure for Defect Mitigation

As previously explained, defects formed during the PBF-LB/M process can be minimized by process optimization but may not be completely avoided. Intrinsic defects in the PBF-LB/M process, such as lack of fusion, gas porosity and unmelted particles, are critical for fatigue, as crack nucleation usually takes place at stress concentration points. In order to achieve a near-perfect level of densification and thus obtain mechanical performance suitable for the high-criticality applications, post-processing techniques for defect mitigation have taken hold in the PBF-LB/M field in recent years. A performing method widely used in powder metallurgy and cast metals is the HIP. During the HIP process, the part is submerged in inert atmosphere (Ar) and simultaneously subjected to high temperature and confining gas pressure [118]. The temperature is set to reduce the yield strength below the gas pressure, thus allowing plastic flow, while high pressure causes substantial shrinkage or complete closure of pores [119]. Considering the efficiency of the HIP in closing pores with a diameter up to 5 mm in Ti6Al4V casting samples and the fatigue strength improvement of about 50% over the non-HIPped specimens recorded in an A356 casting sample, the application of this method was extended to the PBF-LB/M sector [120,121]. On AlSi10Mg samples produced by PBF-LB/M, an impressive decrease in the porosity volume fraction of about 64% was observed as a consequence of HIP [122]. Although the porosity could be significantly reduced with this technique, some critical aspects could be identified:

- The effect of HIP on fatigue behaviour of the final component
- Surface defect formation
- Pore opening and blistering effect after heat treatment

Starting from the influence of HIP treatment on the fatigue proprieties, a strong relation with the HIP parameters needs to be highlighted. Indeed, the temperature values typically used during HIP treatment are higher than the alloy solubility temperature, resulting in critical microstructural changes. The high temperatures may lead to a microstructural coarsening that compromises the fine microstructure typical of PBF-LB/M by dissolving grain boundaries and thus worsening the fatigue behaviour of the final component. Notwithstanding this, Schneller et al. [122] demonstrated that the adverse effects of coarse HIP microstructure could be counteracted by performing HIP treatment at a temperature commonly used for solution annealing followed by a low-temperature annealing heat treatment. Following this innovative treatment programme, they recorded a 14% improvement in high-cycle fatigue strength with a component survival probability of +50% [122]. Looking more closely at the second critical aspect identified, it was noted that the HIP process could cause a break-through and opening of near-surface pores, leaving surface defects known as notches [123]. The force that acts of the surface allows the penetration of Ar into the near-surface pores. This results in a significant reduction of the pore closure effectiveness. Lastly, pore opening and blistering effect were observed when the annealing treatment was performed after the HIP process [124]. A possible explanation could be that during the HIP process, the pores which are filled with argon gas remain closed at high-pressure. However, when the specimen is heat treated at high temperature, the gas trapped expands by enlarging again the internal pore. If the pore is close to the surface the expansion of the gas can break the thin layer of metal, causing a blistering effect.

### 4.3. Heat Treatments

**Figure 10:**
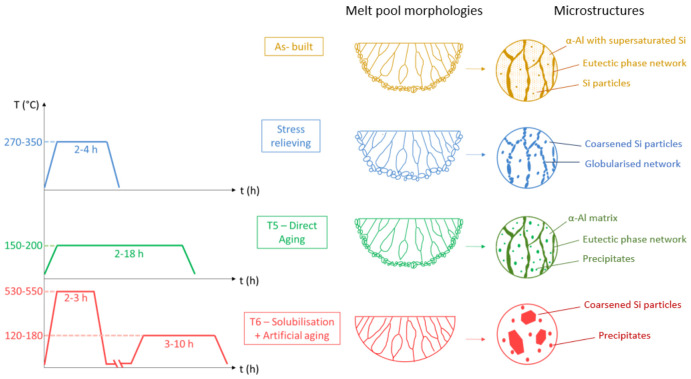
Thermal post-processing and its effects on melt pool morphology and microstructure in Al–Si alloys.

The high cooling rate typical of the PBF-LB/M process results in an extremely fine non-equilibrium microstructure compared to conventional processes. This allows the grain refining strengthening to be exploited, resulting in high mechanical performance already from the as-built state. Moreover, as can be observed from the yellow schematisation in Figure 10, the peculiar microstructure of Al-Si alloys in the as-built state is characterised by a fine eutectic phase network, a matrix of α-Al with supersaturated Si and some fine and uniformly dispersed Si particles. This condition allows synergistic exploitation of solid solution and precipitation reinforcement in addition to grain refinement strengthening. However, the main weakness of the high cooling rate lies in the generation of considerable residual stresses in the as-built components. The need to decrease the tensional state of the built samples led to applying stress-relieving treatments after the PBF-LB/M process [125]. In addition, to increase the strengthening of PBF-LB/M alloys, different thermal post-processing treatments are often performed. The heat treatments generally adopted for Al-Si alloys are artificial ageing treatments such as the T5 treatment-direct ageing and the T6 treatment, which consists of a combination of solubilisation and ageing. These treatments and their effects on melt pool morphology and microstructure of Al-Si systems have been illustrated in Figure 10.

As reported in blue in Figure 10, the stress relieving treatments for Al-based alloys usually involve temperatures in the 270–350 °C range maintained for 2–4 h. Although this allows the component residual stresses to be significantly reduced, the grains result coarsened, the eutectic phase network globalised and the Si particles grown [126]. Up to now, no stress-relieving treatment has been found that does not compromise all the beneficial effects of the as-built microstructure of the PBF-LB/M process. One approach attempted in the literature was to adjust the composition of aluminium base alloys by adding alloying elements that induce the precipitation of intermetallic phase with a strong coarsening resistance [127]. Designing alloys that can age at higher temperatures, close to the stress relief temperature of 300 °C, would allow stress relief and strengthening goals to be achieved with single high-temperature ageing treatment. For example, this approach led to the patenting of Scalmalloy. The addition of slow-dissolving Sc and Zr to the non-heat-treatable Al–Mg system has, in fact, been demonstrated to induce impressive age hardening, achieving excellent stress-relieving and strengthening results with a simple post-heat treatment at 300–400 °C [128]. However, due to the high cost of these alloying elements, it is necessary to continue researching and developing tailored stress-relieving treatments for non-heat-treatable Al-based alloys without implying a change in the alloy formulation also on standard composition.

As above mentioned, in the as-built state there is a favourable condition of supersaturated solid solution. The high PBF-LB/M cooling rate has, in fact, the added advantage of increasing the amount of solute in solution even for elements with low solubility in aluminium. In particular, it was proved that this amount in Al–Si–Mg compositions processed via PBF-LB/M may be higher than that achievable with solubilisation treatment and quenching [129]. This condition in the as-built state can potentially make the PBF-LB/M Al-based alloys suitable for a fast and high performing T5—direct ageing treatment. Starting from the supersaturated solid solution, the strengthening could be achieved with artificial ageing through the precipitation of coherent and semi-coherent nanometric particles. Strengthening by precipitation occurs due to three main phenomena: lattice distortion, shear resistance by dislocations and the Orowan mechanism [130,131]. Among the artificial ageing treatments, T5 is desirable as it avoids the solubilisation treatment required in T6. The solubilisation causes significant grain coarsening, thus losing the benefits of the fine microstructure obtained through rapid solidification [132]. Furthermore, the T6 treatment on Al-Si alloys leads to the disappearance of the melt pool morphology typical of the as-built state and results in a microstructure with no eutectic networks, coarsened Si particles and precipitates of different phases, according to the schematic representation plotted in red in Figure 10. However, T6 treatment is not always avoidable as the PBF-LB/M layer-by-layer construction involves successive heating and cooling cycles with consequent local phase transformations and precipitation. In some cases, this condition leads to the necessity to perform T6 to homogenise the microstructure with the solubilisation treatment and then proceed to the artificial ageing [133]. In addition, the T5 treatment is not always advisable for its detrimental effects of component properties anisotropy and reduced ductility. These side effects of direct ageing treatments are poorly discussed in the literature. This lack of literature could be explained as the effect of heat treatments strongly depending on the chemical composition of the Al-based alloys and the PBF-LB/M process conditions. To date, the standardisation of heat treatments is a long way off, and these must be studied on a case-by-case basis, resulting in a highly time-consuming and powder-intensive procedure.

### 4.4. Surface Post-Treatments

**Figure 11:**
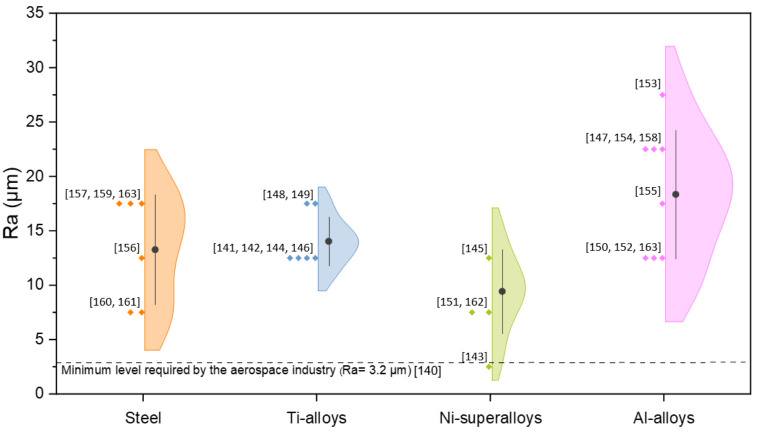
Plot of surface roughness as a function of the main categories of commercially available materials for PBF-LB/M production [130,131,132,133,134,135,136,137,138,139,140,141,142,143,144,145,146,147,148,149,150,151,152,153,154,155,156,157,158,159,160,161,162,163].

The metallic parts produced via PBF-LB/M are characterised by several irregularities that are caused by the layer-by-layer nature of the additive process and the complex physical phenomena occurring during the deposition and fusion of the material [104]. The effect of process parameters on surface roughness of metallic samples produced via PBF-LB/M was already stated in literature. In particular, Yadroitsev et al. [134] obtained smoother surfaces accurately optimising hatch distance and layer thickness. Li et al. [105] revealed that also the scanning direction, the gas flow orientation, and the recoater movement play an important role in part roughness. In addition, Maleki et al. [106] stated that the part geometry, surface orientation with respect to the build direction and the use of support structures may highly affect the surface quality. However, although surface roughness can be limited with careful optimisation of process parameters, the average PBF-LB/M roughness is always above the 3.2 µm required in aerospace applications, regardless of the alloy used [135,136]. Reviewing the widely used material categories in the PBF-LB/M process, it was evident that aluminium alloys record the highest mean surface roughness (Figure 11). This result makes the surface post-treatments research even more felt for the development of Al-based alloys.

In order to avoid premature failure from surface-initiated cracking, PBF-LB/M processed parts in Al-based alloys generally require finishing operations. A wide variety of surface post-treatments have been exploited in literature and an exhaustive review classified them into four main categories [161]:

- Material removal;
- No material removal;
- Coating;
- Hybrid treatments.

More than 25 different treatments fall into the first three categories and their combinations are countless. Among all the possibilities of surface post-treatment, mechanical surface treatments, such as shot peening (SP), ultrasonic nanocrystalline surface modification (UNSM) and chemical surface treatments, such as electro-chemical polishing (ECP) are often reported in the literature as the best performing for components produced by PBF-LB/M [107,162]. ECP is a “material removal process”, while SP and UNSM are “no material removal” treatments that involve severe surface plastic deformation to modify the surface morphology, improving the functionality and lifetime of mechanical components. The remarkable positive effects that can be achieved with these treatments are due to the induced grain refinement and the residual compressive stresses generated in the plastically deformed surface layer.

**Figure 12:**
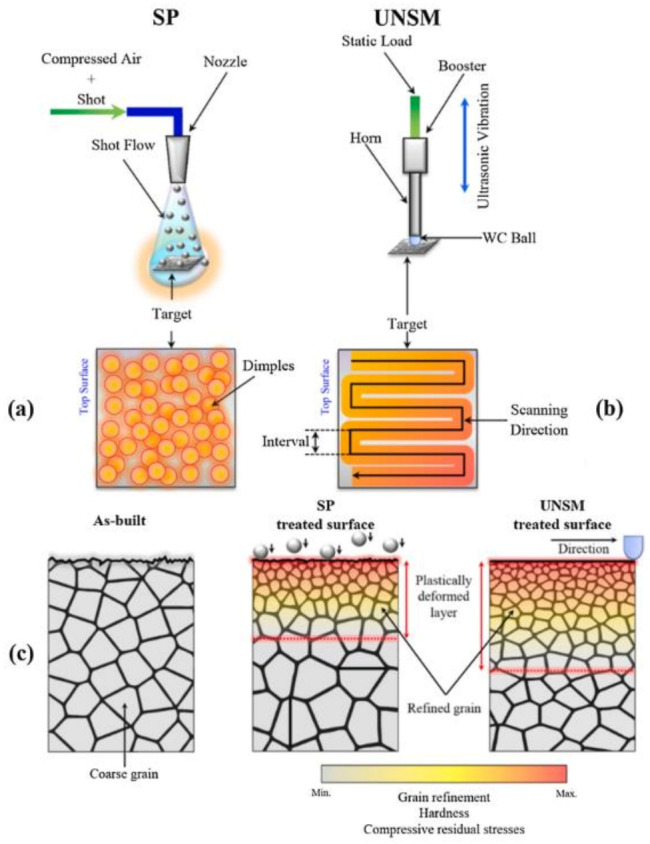
Schematic representation of the SP (**a**) and UNSM (**b**) processes (**c**) and how they affect the surface morphology, grain refinement, surface hardening and residual compressive stresses induced in the surface layer [102].

Looking more closely at the SP treatment, as reported in Figure 12, it is a cold working process in which the surface of a material is bombarded with a stream of small shots under controlled conditions, such as shot size and pressure [107]. Musekamp et al. [153] demonstrated the efficiency of SP in removing the powder particles and agglomerations in Scalmalloy samples, resulting in a higher fatigue strength compared to turned specimens with lower surface roughness. However, as Subramaniyan et al. [163] observed, this technique also exhibits weaknesses, such as the possible appearance of undesirable micro-cracks, craters and delamination on the surface due to imparted kinetic energy. To overcome this issue, some have explored the synergetic use of SP and ECP to improve the fatigue strength and corrosion behaviour of final components [162,164]. In fact, the use of a “material removal” process as the ECP treatment on shot peened samples allowed the surface irregularities to be mostly removed. Another surface post-treatment frequently used alone or in combination with other approaches to perform a hybrid treatment is the UNSM treatment. As well explained by Maleki et al. [161] and illustrated in Figure 12, the UNSM process involves repetitive impacts of a tungsten carbide tip at ultrasonic frequencies under a controlled static load are used to reduce the surface roughness. The UNSM treatment proved to have a higher effect on surface layer hardening and inducing compressive residual stresses with respect to the SP treatment on AlSi10Mg samples [107]. Despite the excellent performance individually recorded by the two mechanical surface treatments described above, to date, the best fatigue, corrosion and wear behaviour has been obtained with the combination of SP and UNSMN [161]. It is clear from these preliminary investigations that the new frontier in surface treatments is hybrid treatments aimed at maximising the performance of individual processes and obtaining high-performance components with surfaces suitable for the most extreme applications. However, there are limited available standards to help researchers and companies approaching this type of surface treatment and less than 1% of ISO/ASTM standards are currently related to surface finishing. Therefore, the research and standardisation work in this field still requires a lot of time and effort to be completed.

## 5. Conclusions

The aim of the present paper was to explore all the challenging aspects in the PBF-LB/M processing of Al-based alloys in order to critically examine the solutions already proposed in the literature, suggest novel approaches and extrapolate which challenges remain to be addressed. Three phases of the Al-based alloy development were investigated: pre, during and post-PBF-LB/M processing. Looking more closely at the pre-processing operations, the main challenges can be briefly summarised as follows:

(1) Design of novel compositions:
  (a) Developing of analytical and computational models to provide a time and cost-saving approach for designing and optimising alloy compositions;
  (b) The availability of the thermophysical data underlying the simulations;
  (c) The model selection for the simulation of solidification mechanisms in PBF-LB/M processes;
  (d) The transition from merely predicting the phases formed during the additive process to predicting their impact on the microstructure;
  (e) Reducing the high degree of uncertainty of these models in the prediction of processability and mechanical properties.
(2) Powder characterisations:
  (a) The absence of a univocal standard for the characterisation of AM powders;
  (b) Quantifying suitable PSD and powder morphology to ensure a proper trade-off between flowability and packing properties;
  (c) Studying and sharing the effect of atomisation parameters on powder properties;
  (d) Understanding the factors with the strongest influence on powder reuse to carry out repeatable and quantifiable studies.
(3) Process parameters optimisation:
  (a) Implementing a univocal rule for calculating the optimum layer thickness considering the thermophysical properties, the characteristics of the powder and the PBF-LB/M machine features

The main critical aspects that could be faced during Al-based alloy processing are:

(1) The poor absorbance of powders at YAG laser wavelengths. This problem is usually overcome by using very high VEDs. However, a solution that is still rarely used in the literature but for which there are several industrial patents is the use of green lasers.
(2) The generation of hot tears and/or cracks and delamination. These problems are often solved by acting on the chemical composition or some process conditions, such as using heated platforms or support structures.
(3) The heterogeneous and anisotropic microstructure caused by the directional thermal flow and rapid cooling rates involved in the PBF-LB/M process. The microstructure anisotropy negatively affects the mechanical behaviour of the Al-based parts, especially if multi-axial loads are applied.
(4) The formation of PBF-LB/M typical pores that may have a detrimental effect on the mechanical properties of the final component.

To act on all the above mentioned critical aspects, it is usually necessary to change the process conditions. To minimise trial-and-error experimentation, overcome the limitations of simulation systems and increase the repeatability of PBF-LB/M processes, a powerful all-encompassing solution could be using data from in-situ monitoring as input for machine learning techniques. However, the development of this loop process still requires numerous studies.

Looking more closely at the post-processing operations, four critical aspects were investigated:

(1) Defect investigation methodologies with their benefits and disadvantages.
(2) Porosity closure techniques such as HIP. In particular, two HIP weaknesses were identified:
  (a) The possible breaking and opening of near-surface pores, leaving surface defects known as notches.
  (b) The opening of pores and the blister effect that can occur when heat treatments are performed after the HIP process.
(3) The development of heat treatments that relieve residual stresses and decrease microstructural anisotropy without, however, deteriorating the typical microstructure of the PBF-LB/M process or drastically reducing the ductility of the samples.
(4) Providing and designing surface treatments that make the sample roughness suitable for more restrictive applications, such as in the aerospace industry.

This paper has highlighted that although great progress has been made in achieving a better scientific understanding of Al-based alloy processing via PBF-LB/M, many critical challenges still need to be addressed. This review demonstrates that knowledge of the weaknesses of these alloys and this additive process are the starting point for the development of solutions that break down all barriers and make Al alloys processed by PBF-LB/M competitive even on an industrial level.

## Figures and Tables

**Table 1:** Maximum thicknesses producing low signal-to-noise ratios related to X-ray voltage.

Materials	*X-ray Voltage*				
	130 kV	150 kV	190 kV	225 kV	450 kV
Steel\Ceramic	5 mm	<8 mm	<25 mm	<40 mm	<70 mm
Aluminium	<30 mm	<50 mm	<90 mm	<150 mm	<250 mm
Plastic	<90 mm	<130 mm	<200 mm	<250 mm	<450 mm
